# Melatonin ameliorates disease severity in a mouse model of multiple sclerosis by modulating the kynurenine pathway

**DOI:** 10.1038/s41598-022-20164-0

**Published:** 2022-09-24

**Authors:** Yahya Jand, Mohammad Hossein Ghahremani, Amir Ghanbari, Shahram Ejtemaei-Mehr, Gilles J. Guillemin, Mahmoud Ghazi-Khansari

**Affiliations:** 1 Department of Pharmacology, School of Medicine, Tehran University of Medical Sciences, P.O. Box 13145-784, Tehran, Iran; 2 Department of Pharmacology and Toxicology, Faculty of Pharmacy, University of Medical Sciences, Tehran, Iran; 3 Cellular and Molecular Research Center, Yasuj University of Medical Sciences, Yasuj, Iran; 4 Neuroinflammation Group, Faculty of Medicine and Health Sciences, Macquarie University, Sydney, NSW Australia

**Keywords:** Neuroimmunology, Molecular neuroscience, Multiple sclerosis

## Abstract

Melatonin (MT), a neurohormone with immunomodulatory properties, is one of the metabolites produced in the brain from tryptophan (TRP) that has already strong links with the neuropathogenesis of Multiple sclerosis (MS). However, the exact molecular mechanisms behind that are not fully understood. There is some evidence showing that MS and MT are interconnected via different pathways: Relapses of MS has a direct correlation with a low level of MT secretion and a growing body of evidence suggest that MT be therapeutic in Experimental Autoimmune Encephalomyelitis (EAE, a recognise animal model of MS) severity. Previous studies have demonstrated that the kynurenine pathway (KP), the main pathway of TRP catabolism, plays a key role in the pathogenesis of MS in humans and in EAE. The present study aimed to investigate whether MT can improve clinical signs in the EAE model by modulating the KP. C57BL/6 mice were induced with EAE and received different doses of MT. Then the onset and severity of EAE clinical symptoms were recorded. Two biological factors, aryl hydrocarbon receptor (AhR) and NAD^+^ which closely interact in the KP were also assessed. The results indicated that MT treatment at all tested doses significantly decrease the EAE clinical scores and the number of demyelinating plaques. Furthermore, MT treatment reduced the mRNA expression of the KP regulatory enzyme indoleamine 2,3-dioxygenase 1(IDO-1) and other KP enzymes. We also found that MT treatment reduces the mRNA expression of the AhR and inhibits the enzyme Nicotinamide N-Methyltransferase (Nnmt) overexpression leading to an increase in NAD^+^ levels. Collectively, this study suggests that MT treatment may significantly attenuates the severity of EAE by altering the KP, AhR and NAD^+^ metabolism.

## Introduction

Multiple sclerosis (MS) is a chronic and progressive inflammatory disease characterized by demyelination and inflammation in the central nervous system. It has an unclear multifactorial aetiology and many biochemical, immunological, and environmental causative factors have been proposed^1–4^. There is already significant evidence suggesting that the kynurenine pathway (KP), the main catabolic pathway of tryptophan (TRP), has a strong association with the immune system and plays crucial roles in the development of MS and other neurodegenerative diseases^5–8^. In 1979, Monaco et al. showed that the level of TRP in the cerebrospinal fluid of MS patients is lower than that of control subjects^9^. Also, the same research demonstrated that the ratio of kynurenine/tryptophan in MS patients is higher than in control subjects, which indirectly indicates IDO-1 enzyme activation^10^.

In physiological conditions, TRP is catabolised along two main pathways: (1) Serotonin/melatonin and (2) the KP/ NAD^+^. In inflammatory conditions, > 95% of TRP is processed by the KP leading to the production of several neuro- and immune-active metabolites. The important enzymes in the KP are indoleamine 2,3-dioxygenase 1, 2 (IDO-1, 2), tryptophan 2,3-dioxygenase (TDO), kynurenine monooxygenase (KMO) and kynurenine aminotransferases (KATs). Degradation of TRP through the KP leads to the formation of metabolites such as quinolinic acid (QUIN) and kynurenic acid (KA) which has neurotoxic and neuroprotective properties, respectively^10^. Prior research in this area has shown that the activity of the KP in MS patients is significantly different from that in healthy subjects^6^. For instance, the activity of IDO-1 and KMO is increased in MS patients and inhibition of these enzymes can ameliorate the severity of MS^11^. Furthermore, the activity of KAT 1 and 2 are increased in serum and red blood cells of MS patients^12^. Furthermore, Lanz et al. demonstrated that both expression and activity of TDO are increased in the liver of Experimental Autoimmune Encephalomyelitis (EAE) mice^13^.

MT is another metabolite of TRP, and recently a growing body of evidence has demonstrated its anti-inflammatory, immunomodulatory, and other beneficial biological effects^14–17^. MT secretion from the pineal gland follows a circadian rhythm and light inhibits its secretion. Epidemiological studies also showed evidence of a link between MT and MS. The disease is more prevalent in high latitudes and pieces of evidence also have shown that the flares of MS are more frequent in spring and summer. In other words, it can be speculated that the length of daytime (less MT produced) has a strong direct correlation with MS incidents and flare-ups^18,19^. Based on these findings, the hypothesis of a link between MT and MS was formed and became more compelling when other studies demonstrated that sleep abnormalities are more prevalent in MS patients. Furthermore, in accordance with the evidence mentioned, different animal studies have shown that MT can alter EAE through the modulation of various pathways^2,20–22^.

The over-expression of the enzyme Nicotinamide N-Methyltransferase (Nnmt) leads to higher catabolism of NAD^+^, an essential co-factor for the maintenance of the energetic balance in cells. In fact, NAD^+^ depletion may play a key role in the pathogenesis of axonal injury and neurodegeneration in MS^23–25^. Prior studies have shown that the level of NAD^+^ and the NAD^+^\NADH ratio were lower in serum of MS patients in comparison with healthy subjects^26,27^. As de novo synthesis is the main source of NAD^+^ in macrophage cells, and MT can affect the KP along with increasing the NAD^+^\NADH ratio^28,29^, the decrease in the degradation of NAD^+^ could be one of the mechanisms for the protective effect of MT on EAE. This study aimed to assess the roles of the KP, the AhR and NAD^+^ in EAE animals treated with different doses of MT.

## Results

### Melatonin lowers EAE clinical score

**Figure 1:**
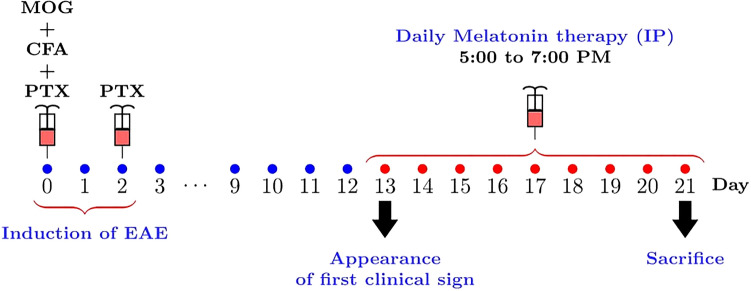
Schematic representation of the experimental procedures. EAE was induced in C57BL6 mice (day 0) that were treated at day 13 with various doses of melatonin until day 21. Daily clinical scores were then measured, which continued until day 21.

**Figure 2:**
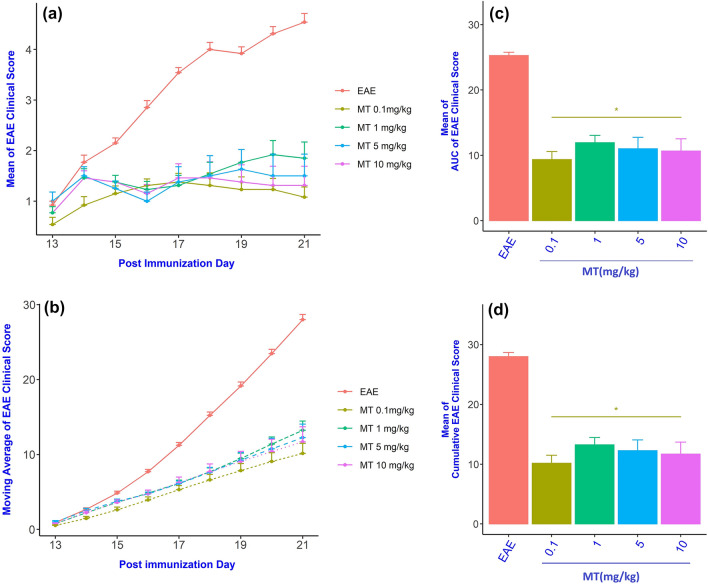
Amelioration of EAE in melatonin treated mice. Analysis of neurological disability displayed: (**a**) Dailly clinical score, (**b**) Moving average of the daily clinical score, (**c**) AUC of the daily clinical score and (**d**) Cumulative neurological disability, were significantly lower in melatonin compare to PBS treated EAE mice. Data are expressed as the Mean ± SEM (n = 8–13). Statistical analysis was performed by one-way analysis of variance (ANOVA) followed by the Tamhane test. Significance is indicated by *p < 0.0001 vs. EAE.

To assess the effect of MT on the severity of EAE, MT was administered by daily i.p. injections in various doses, starting from day 13 after immunization until the end of the study on day 21. All of the animals were alive at the end of study. The first ameliorating effect of MT was observed on day 15 and continued until day 21, as assessed by the clinical score (Fig. 2a,b). To compare the clinical scores of each group in the timeline of the study, we calculated the area under the curve (AUC) of clinical scores and compared this parameter between groups with one-way ANOVA. (Fig. 1, supplementary materials).

The AUCs of the clinical scores were significantly different between groups, Welch’s F[4,23.45] = 64.58, *p* < 0.001). As shown in Fig. 2c, 0.1 mg/kg of MT significantly decrease the AUC. Comparing different treatment groups (0.1, 1.0, 5.0, 10 mg/kg) using the Tamhane test as a Post Hoc test showed that there was no difference between groups that were treated with MT *p* > 0.05, (Fig. 2c; Table 2 in supplementary material).

One-way ANOVA of cumulative clinical scores revealed that cumulative clinical scores were significantly different between groups, Welch’s F[4,23.26] = 66.58, *p* < 0.001). Figure 2d shows that 0.1 mg/kg of MT significantly decrease the cumulative clinical scores. Comparing different treatment groups (0.1, 1.0, 5.0, 10 mg/kg) using the Tamhane test as a Post Hoc test, showed that there was no difference between groups that were treated with MT *p* > 0.05, (Fig. 2d; Table 3 in supplementary material).

### Melatonin reduces the number of demyelinating plaques

**Figure 3:**
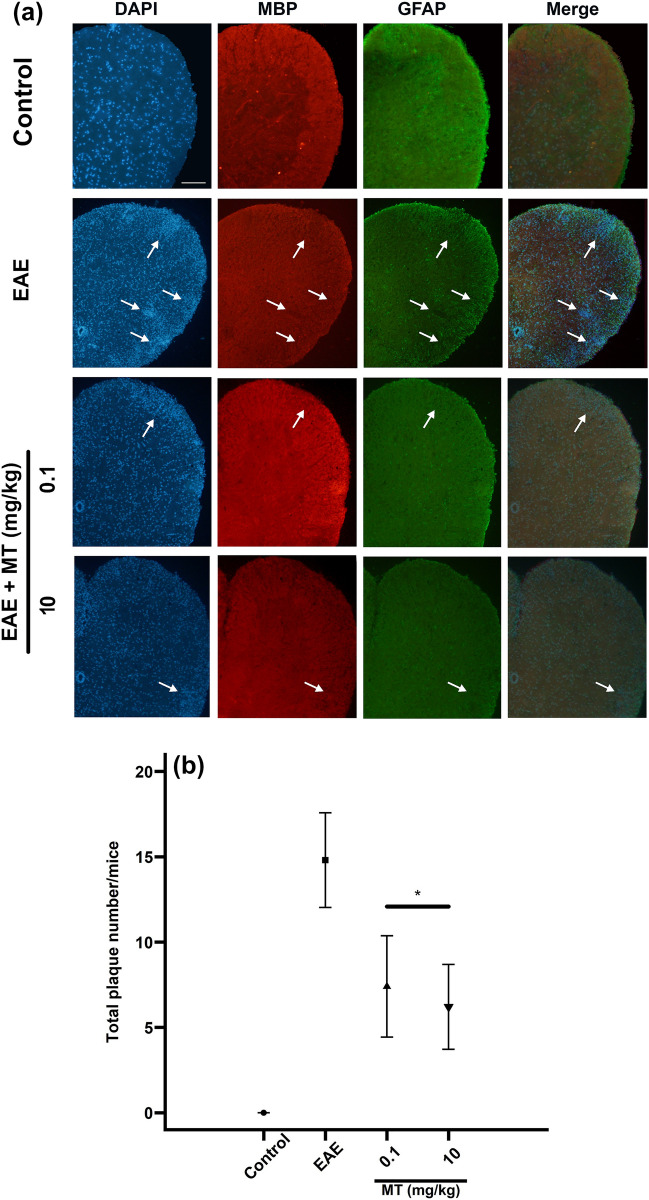
Effect of melatonin on glial and oligodendrocyte cells. Representative immunofluorescence images of astrocytes (GFAP positive cells, green) stained with anti-GFAP and anti- MBP (Red) and Hoechst (a nuclei marker, blue) in control, and treated groups at day 21. (**a**) Accumulation of GFAP positive cells along with decreased in MBP positive stained area displayed demyelination plaques in EAE group while in MT treated mice increased MBP positive stained area along with decreased in astrocytes (remyelination) was seen which indicated that MT can prevent formation of demyelination plaque in EAE mice. (**b**) Quantification of total plague number in the spinal cord. Melatonin treated EAE mice have less plaques than PBS treated EAE mice. Each group included 3 replicates (n = 3). Values are presented as mean ± SEM for 15 sections. Statistical analyses were performed by the ANOVA followed by the Tukey test. Significance is indicated by *p < 0.001 vs. EAE. Scale bar,100 µm.

To assess the induced demyelination in the spinal cord, we used immunohistochemical staining for Myelin basic protein (MBP) and Glial Fibrillary Acidic Protein (GFAP) (Fig. 3a). Our data showed MT can significantly decrease the number of demyelinating plaques F[3, 16] = 184.33,*p* < 0.0001. Comparing groups using Tukey’s test as a Post Hoc test, showed that the number of plaques in the EAE group has significantly more than treated groups *p* < 0.001, (Fig. 3b).

### Melatonin downregulates the kynurenine pathway activity

**Figure 4:**
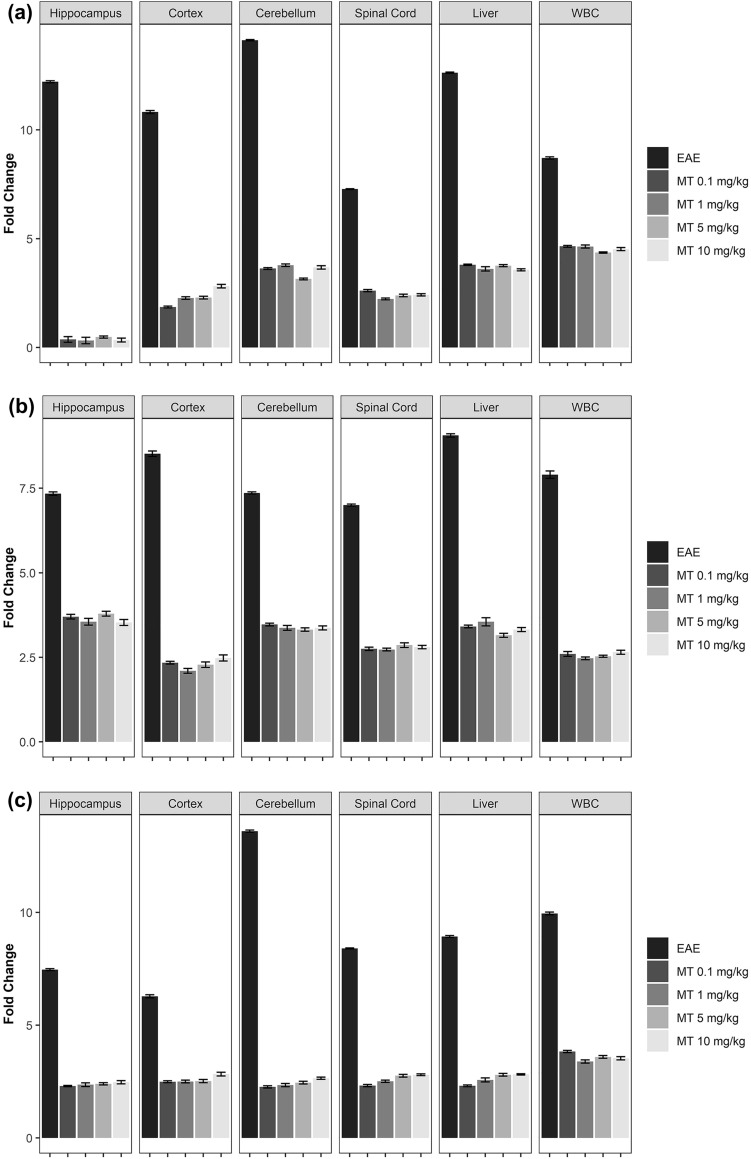

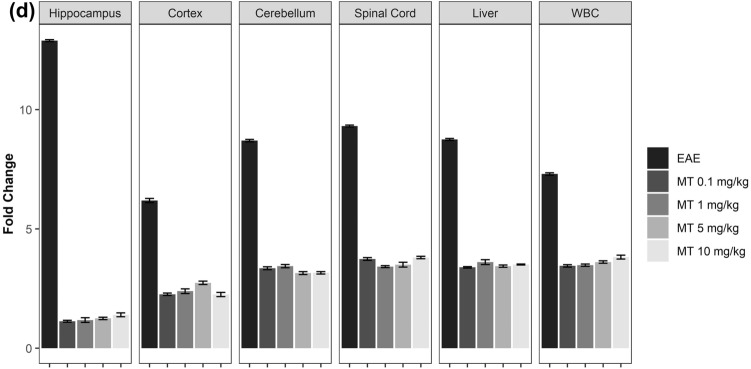
Effect of melatonin on mRNA expression of KP in different part of brain (Hippocampus, Cortex, Cerebellum), Spinal Cord, Liver and WBC. mRNA expression level of (**a**) IDO-1, (**b**) IDO-2, (**c**) TDO, (**d**) KMO were determined by qPCR. B2M and Eef1e1 were used as internal control. Quantification of mRNA expression levels was normalized to controls. Values are presented as a fold change compared to the control group.

**Figure 5:**
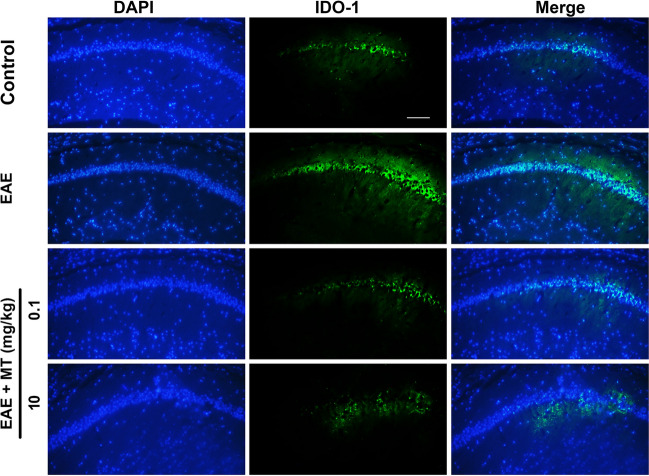
Effects of melatonin on IDO-1 protein expression in EAE mice. Representative images of Immunofluorescence staining showed that IDO-1 protein (green) is expressed in hippocampus area of EAE mice. The hippocampal structure is defined by fluorescent signal derived from DAPI positive cell nuclei (Blue). Scale bar, 25 µm.

**Figure 6:**
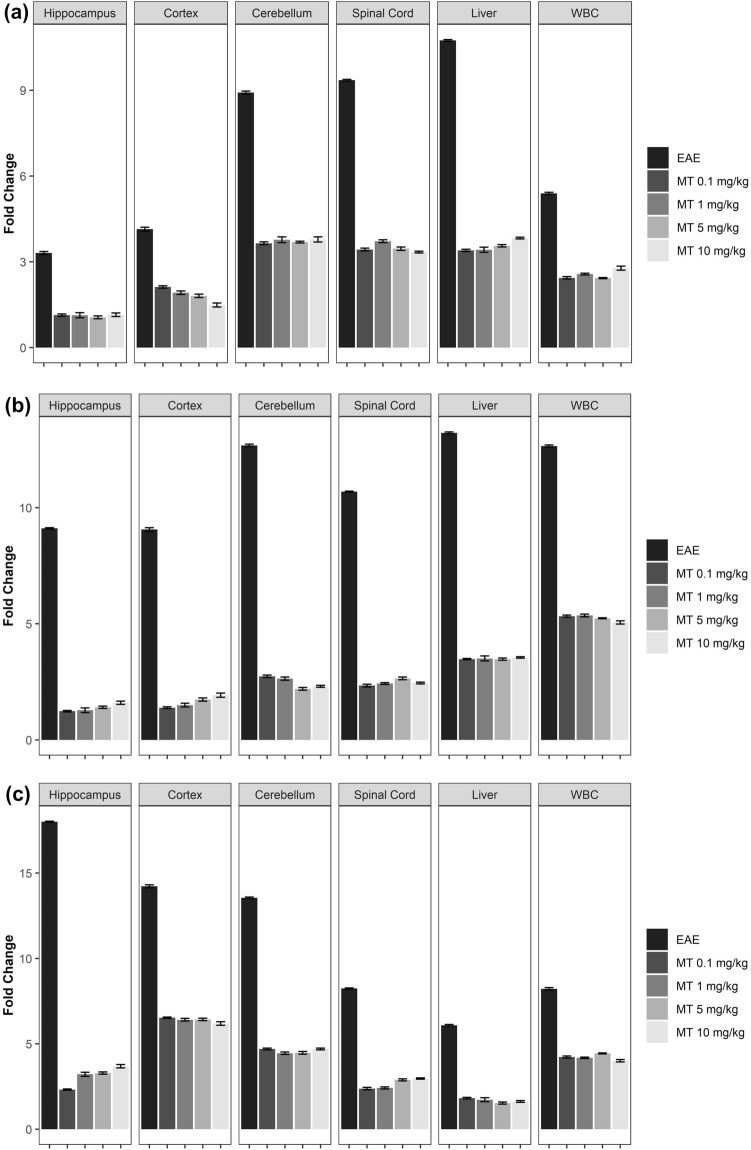
Effect of melatonin on mRNA expression of selected genes in different part of brain (Hippocampus, Cortex, Cerebellum), Spinal Cord, Liver and WBC. mRNA expression level of (**a**) AhR, (**b**) Interferon gamma, (**c**) Nnmt, were determined by qPCR. B2M and Eef1e1 were used as internal control. Quantification of mRNA expression levels was normalized to controls. Values are presented as a fold change compared to the control group.

To assess the effects of MT on the kynurenine pathway in EAE, we used qPCR. EAE increased the mRNA expression of KP enzymes. Furthermore, MT significantly and dose-independently reduced the expression of these genes. (Fig. 4a–d). In addition, the immunohistochemical staining of hippocampus in EAE mice showed expression of IDO-1 protein in this area. (Fig. 5). Furthermore, our findings indicated that melatonin could decrease the expression of AhR and Interferon gamma’s mRNA (Fig. 6a,b).

### Melatonin decreases the catabolism of NAD^+^

To assess the effect of melatonin on the consumption of NAD^+^ in EAE, we measured the expression of mRNA for Nnmt, the enzyme that metabolizes NAD^+^, mRNA. Our results suggested that EAE can significantly induce the expression of Nnmt mRNA while melatonin reduces it (Fig. 6c).

## Discussion

We evaluated the effects of MT on the KP in the EAE model. Our data indicate that MT at all the doses tested can alleviate EAE Severity by attenuating neuroinflammation, decreasing the expression of the KP enzymes, the AhR and Nnmt enzyme. The inhibitory effect of MT on TDO, observed in our study, is in accordance with the results of the Walsh et al*.* study, showing that both MT and serotonin can inhibit TDO activity^30^. Our finding shows that MT at dose of 0.1 mg/kg had the same response as higher doses (1.0, 5.0, 10 mg/kg). It seems MT at dose of 0.1 mg/kg may saturate the receptors thereby increases the doses had no additional effects (Fig. 2c,d).

There is a growing body of evidence showing a correlation between levels of MT and MS. On one hand, epidemiological studies have shown that MS incidence is increased in higher latitudes and specific seasons (spring and summer), the situations correlated with lower MT’s level^18,19^. On the other hand, it is well established that MT has potent anti-inflammatory and immunomodulatory activity. Previous studies have shown that the MT administration can ameliorate the severity of EAE^20–22^. Our present data extend the results of our previous study, which showed that MT can ameliorate the severity of EAE^2^. Additionally, our findings indicate that the expression of KP enzymes are increased in EAE and that MT can decrease them. Activation of IDO-1 and TDO is a double-edged sword with respect to the immune system and neurodegenerative diseases^31^. Activation of these KP enzymes enhances antibacterial activity of the immune system^32^, but on the other hand, increases KP metabolites such as KYN, QUIN, and 3-HAA, which alongside tryptophan starvation, can lead to immune-suppression by triggering apoptosis in TH_1_ cells. This will facilitate suppression of the immune system through increased T_reg_ cells as seen in cancer and chronic infections states^33–36^.

Chronic activation of IDO-1 leads to subsequent activation of other enzymes producing neuroactive metabolites that can affect the severity of neurodegenerative diseases. Neuroactive metabolites of this pathway can be both neurotoxic such as QUIN and neuroprotective, such as KYNA. The main mechanism for the neuroprotective effect of KYNA is through the antagonistic activity of NMDA, by antagonising the excitotoxic effect of QUIN. Another important neuroprotective property of KYNA is its antioxidant activity^37^. On the contrary, some studies have shown that KYNA can impair memory and reduce dopaminergic and glutaminergic neurotransmission^38^. Accumulation of KYNA in the presynaptic space causes postsynaptic inhibition of NMDA receptors, nicotinic acetylcholine receptors, and AMPA receptor-mediated current. The neurotoxic mechanisms of QUIN are numerous^39^. For example, QUIN is an NMDA receptor agonist and causes neuronal damage through intermittent receptor stimulation leading to impaired calcium homeostasis, increasing oxidative stress and triggering mitochondrial damage. These changes are usually associated with programmed cell death. In addition, the production of QUIN-iron complex increases reactive oxygen species (ROS), lipid peroxidation and ultimately DNA damage. Furthermore, in physiological conditions, the KP is one of the pathways involved in the de novo synthesis of NAD^+^, and macrophages supply their NAD^+^ pool, mainly through this pathway. QUIN is the substrate for QPRT, the KP enzyme for the production of NAD^+^; but because the QUIN-producing enzyme is eighty times more active than its catabolizing enzyme, the production of QUIN is always greater than its conversion to NAD^+^, which leads to the accumulation of QUIN^40,41^. QUIN can also alter redox homeostasis by reducing glutathione and Cu–Zn-SOD levels. therefore, chronic activation of the KP is likely to be harmful to cells^5,6,33,42^. As we found that MT reduces the expression of all the KP enzymes, we can speculate that MT slows down the KP, leading to a reduction in the level of neurotoxic metabolites and subsequently alleviating the severity of EAE.

In our study, we showed that AhR expression is increased in EAE. The results of our study are in accordance with another study showing that activation of AhR was associated with the development of EAE^43^. Some studies have shown that activation of AhR by KYNA can synergistically induce IL-6 production and activate TH_17_ response^44^. In contrast, some studies reported that activation of AhR by kynurenine increases the expression and activity of IDO-1 and leads to an auto-amplifying loop as more kynurenine is produced and promotes AhR activity^33^. This positive loop will result in the modulation of the immune system balance between T_reg_ and TH_17_. The effects of AhR activation on the immune system can be diverse, as some studies have shown that FICZ and TCDD (two AhR ligands) can have opposite effects on the immune system; depending on where the ligand bound the AhR^45,46^. Furthermore, AhR activation can induce CYP1A2 and increase the metabolism of MT and subsequently increase the N-acetyl serotonin (NAS)/MT ratio which alone can activate Tyrosine Kinase B (TrkB)^47^. Colombo et al., have shown that activation of TrkB has detrimental effects on EAE^48^. Therefore, inhibition of AhR by MT can decrease TrkB activation by  decreasing nc the NAS/MT ratio^49^. We show in this study that MT can decrease the expression of AhR mRNA and concomitantly IDO-1 mRNA and protein. We hypothesise that in EAE, activation of AhR and IDO-1 have detrimental effects on the immune system, and the mechanism behind MT’s protective effects of MT on EAE is likely the suppression of AhR and IDO-1.

Overactivation of the KP depletes tryptophan from other metabolic pathways using this essential amino acid as a substrate. The outcome is a reduction in the production of other metabolites such as NAS and MT. Previous studies have shown that both metabolites can reduce disease severity in the EAE model and exert their immunomodulatory effects, by binding to the allosteric site of the AhR receptor^33^. MT can increase the activity of Aryl alkylamine N-acetyltransferase (AANAT) which is the enzyme that produces NAS that can be converted to MT. So, another potential mechanism for the beneficial effect of MT in EAE could be the compensatory supplementation of the MT and NAS pools. MT may act as an alternate substrate for IDO-1. So, in the progressive stage of MS, activation of IDO-1 leads to the production of KP neurotoxic metabolites such as QUIN which can be decreased by MT, acting as an alternate substrate.

Previous studies have shown that the level of NAD^+^ is decreased in MS^26,27^, NAD^+^ is such an important co-factor that the human body has three ways to produce it: (1) the KP, (2) the salvage pathway, (3) diet (vitamin B3)^50^. Even if the KP is inhibited by MT, NAD^+^ can still be produced by the 2 alternative sources.

The serum level of NAD^+^ in MS patients is reduced and is correlated with the severity of the disease. Generation of ROS and some lipid breakdown products due to the inflammatory condition of MS leads to DNA damage and the consequent activation of the DNA repairing enzyme Poly ADP-Ribose Polymerase 1 (PARP-1), which catabolise NAD^+^ as the main substrate. PARP-1 overactivation leads to NAD^+^ and ATP depletion^26^. In addition, we showed for the first time, that Nnmt mRNA expression is increased in EAE. Nnmt overexpression will lead to even higher catabolism of NAD^+^ and a higher loss of energy in EAE. Our data showed that MT can reduce Nnmt overexpression in EAE, that represent a new beneficial mechanism of MT on EAE severity.

### Conclusion

Previous studies have shown that MT can alleviate the severity of EAE. Our present findings report a new mechanism of action showing that MT can down-regulate the KP in EAE. At the late stage of MS (progressive form), the chronic activation of the KP leads to the production more neurotoxic KP metabolites, especially QUIN. The regulatory effects of MT on the KP are likely to lead to a decrease in neurotoxicity. MT can also decrease the expression of the AhR which could be associated with a decrease in the inflammatory response. Finally, MT can reduce the consumption of NAD^+^ by inhibiting the enzyme Nnmt. Higher availability of NAD^+^ allows DNA and cell damage repair. This hypothesis needs further experimental and clinical studies looking at the interaction of MT, the KP in both the EAE model and MS patients.

## Materials and methods

### Ethics

All procedures were approved by the Research and Ethics Committee of Tehran University of Medical Sciences (TUMS) (IR.TUMS.MEDICINE.REC.1399.126) and complied with ARRIVE guidelines. All methods were performed in accordance with the relevant guidelines and regulations.

### Animals

In this study, sixty-eight female C57BL/6 mice of 6–8 weeks of age and 16–18 g of body weight were purchased from Iran Pasteur Institute (Pasteur’s Institute, Tehran, Iran). Mice were randomly divided to standard cages, with four to five animals per cage. The animals were kept at constant temperature (22 ± 2 °C), humidity (55 ± 10%), and light cycles (12 h light–12 h dark). Mice were given ad libitum access to food and water.

### Induction of EAE

The EAE model was performed in the “Salari Institute of Cognitive and Behavioural Disorders (SICBD)”. The animal model was induced by injection of MOG35-55 (MEVGWYRSPFSRVVHLYRNGK) as is previously described^51^. Briefly, Under the anaesthesia caused by ketamine hydrochloride (50 mg kg − 1; Alfasan, Woerden-Holland) plus Xylazine (5 mg kg^−1^; Alfasan, Woerden-Holland), mice were immunized with 300 µg of myelin oligodendrocytes glycoprotein (MOG35-55; Sigma-Aldrich, United States) dissolved in phosphate-buffered saline (PBS) and emulsified with an equal volume of complete Freund adjuvant (CFA; 400 µg of Mycobacterium tuberculosis; Sigma Co, USA). Additionally, 300 ng of pertussis toxin (List Biological Labs, Campbell, CA, USA) was injected intraperitoneally, into all animals, on days 0 and 2.

### Treatment of animals and clinical evaluation

Mice were randomly divided into six groups as follows: (A) EAE mice treated by phosphate-buffered saline (PBS) (Vehicle) (n = 13); (B) EAE mice which were treated with MT (Sigma Aldrich, United States) 0.1 mg /kg/day (n = 13); (C) EAE mice which were treated with MT (Sigma-Aldrich, United States) 1 mg /kg/day (n = 13); (D) EAE mice which were treated with MT 5 mg /kg/day (n = 8); (E) EAE mice which were treated with MT 10 mg /kg/day (n = 13); (F) PBS-treated mice (Control) (n = 8). To imitate the clinical situations, treatment was started when the first sign of the disease appeared. MT or PBS was administered intraperitoneally (i.p.) for 9 consecutive days (from day 13 to day 21). To resemble the physiological condition, the MT/PBS treatment were performed between 5:00 pm and 7:00 pm. Mice were sacrificed on day 21 when the mean clinical score in the vehicle group was greater than 4. MT was freshly prepared by dissolving it in PBS and 5% dimethyl sulfoxide (DMSO; Sigma-Aldrich, United States), and then administered at the doses described above. All control, vehicle (EAE mice), and experimental groups received the same percentage of 5% DMSO. The experimental procedures are depicted in Fig. 1.

Clinical signs of the disease in mice were evaluated by the expert observer that blind to the treatment condition, from day 10 to day 21 after immunization using a score as follows: 0 = Normal mouse; no obvious signs of disease, 1 = Limp tail or the hind limb weakness but not both, 2 = weakness of the limb and the hind limb, 3 = Partial hind limb paralysis, 4 = complete paralysis of the hind limb, 5 = Moribund state or death by EAE. The mice were weighed every other day after immunization.

### Sample preparation

After 21 days of the experiment, mice were deeply anesthetized and sacrificed with cervical dislocation. Brain, liver, and spleen were quickly removed. Brains and livers were frozen immediately on dry ice and stored at − 80 °C until further tests were performed. For histopathological studies, 3 mice have used. After animal sacrifice, brain and spinal cord of each mouse removed. Three animal brains tissue and spinal cords in each group were immersed in paraformaldehyde overnight, and then fixed tissues were embedded in paraffin. Lumbar spinal cord and hippocampal area of selected tissue were sectioned (5 µm) and randomly 10 sections selected with 300 µm distance between them^52^. Lymphocytes were obtained from the spleen. Briefly, spleens were removed and then washed with cold PBS. Cells were isolated using a 100 µm cell strainer. After removing RBCs with lysis buffer (mixture of ammonium chloride, sodium bicarbonate and EDTA disodium), lymphocytes were isolated and then aliquoted in 5 × 106 cells in ml and were stored at − 80 °C for further evaluations.

### Real-time PCR

Total RNA was extracted with YTzol Pure RNA (Yektatajhiz azma, Tehran, Iran, Cat. No. YT9064) according to the manufacturer's protocol. RNA quantity and purity were determined by NanoDrop™ One/One^C^ Microvolume UV–Vis Spectrophotometer (Thermo Fisher Scientific, USA). Complementary DNA (cDNA) was synthesized using a cDNA synthesis kit (Yektatajhiz azma, Tehran, Iran) according to the manufacturer’s instructions. To avoid damage to cDNA resulting from freezing and thawing, the synthesized cDNA was aliquoted and stored at − 80 °C. The primers which were used are listed in Table 1 in Supplementary Material. The level of mRNA expressions was determined with qPCR using LightCycler 96 System (Roche Applied Science) using RealQ Plus 2 × Master Mix Green (Amplicon, Denmark) as the detection system in a reaction mixture of final 25 µL volume. The PCR conditions were as follows: initial activation at 95 °C for 15 min, then 45 amplification cycles consisting of denaturation at 95 °C for 15 s, annealing at 60 °C for 20 s, and extension at 72 °C for 20 s. The specificity of the PCR products was confirmed by melting curve analysis. The primer efficiencies were then used to determine relative gene expression by the Pfaffl method^53^. Then relative genes expressions were normalised to Eukaryotic Translation Elongation Factor 1 Epsilon 1 (Eef1e1) and Beta-2 microglobulin (B2M), using the geometric averaging method^54,55^. Briefly, we calculated the relative quantity (RQ) of each gene based of Pfaffl method. Relative gene expression is calculated by division of RQ of each gene on RQ’s geometric mean of normalizer genes.

### Immunohistochemistry

Following deparaffinization and hydration, sections were selected randomly from the lumbar spinal cord and hippocampus. All sections were retrieved by pressure cooker and permeabilized by 20% tween for 20 min for fluorescence immunostaining. The nonspecific area was blocked with 0.1% BSA in 0.1% Triton X-100/PBS for 20 min. The sections were incubated overnight at 4^◦^ C with Anti-MBP antibody (1:500, Abcam [ab218011], USA) and Anti-GFAP antibody (1:800, Abcam [ab68428], USA) and Anti-IDO-1 Antibody (1:250, EMD Millipore [MABF850], Germany), as primary antibody, in 0.03% PBS-Triton- × 100 supplemented with 5% normal goat serum (Abcam[ab7481-], USA). The slides were incubated with appropriate secondary antibodies (Alexa Fluor 488 and 566 (1:500, Thermo Fisher Scientific, USA), and Hoechst (1:1000, Abcam [ab228550], USA) for 1 h. Samples were analysed using a fluorescent microscope (Olympus IX-71; Olympus, Tokyo, Japan) equipped with a Canon EOS digital camera. For quantification of plaque number, we used soteriological dissector method for particle number. after preparation of immunohistochemically imaging of spinal cord, two adjust section with mentioned distance compared them (position and plaque size). The plaque with different position were counted. Totally number of each spinal cord plaque calculated for comparison between groups. Area of plaque estimated image J software.

### Statistics

Results are presented as means with error bars that indicate the standard error of the mean (Mean ± SEM). All data (no data were excluded) were collected in a blinded manner and analysed by one-way ANOVA followed by Tamhane multiple comparisons test. All statistical tests were two-sided and the level of significance was set at *P* value < 0.05. Statistical analysis has done by IBM SPSS software (IBM SPSS Statistics for Windows, Version 26.0. Armonk, NY: IBM Corp.) and R version 4.0.2.

## Supplementary Information

Supplementary Information.

## Data Availability

The datasets generated during and/or analysed during the current study are available from the corresponding author on reasonable request.
