# Supplementary material for: Melatonin ameliorates disease severity in a mouse model of multiple sclerosis by modulating the kynurenine pathway
Source: Sci Rep. 2022 Sep 24;12:15963. doi: 10.1038/s41598-022-20164-0 (PMC9509376; doi:10.1038/s41598-022-20164-0)

| Gene | Forward(5'-3') | Reverse (5'-3') |
| --- | --- | --- |
| IDO-1 | CGAGGCTGGCAAAGAATCTC | GACAAACTCACGGACTGGGG |
| IDO-2 | CAGAAGGACCGTTGGAAATC | TGACTGTGTTGCCGAATGG |
| TDO | GCATCGTGTGGTGGTCATC | GGAACTGTAGACTCTGGAAGCC |
| AHR | CACTGACGGATGAAGAAGGAC | TTGGTGCGTATTGGTAGGG |
| KMO | AGTCCTATGCCAATGCGAAG | GCTCCATCACACCCTACAAC |
| QPRT | CTGAAGGTAGAGGTGGAATGTAG | GACACGCTGGGGAATCTG |
| INFgama | TCAGCAACAGCAAGGCGAA | TCTCTTCCCCACCCCGAAT |
| Nnmt | AAACGAGATCCTCAGACATC | CTCCTTTTACAGCACCCAG |
| B2M | GGTCTTTCTGGTGCTTGTCTC | CAGTTCAGTATGTTCGGCTTC |
| Eef1e1 | TCCAGTAAAGAAGACACCCAGA | GACAAAACCAGCGAGACACA |

**Table 1: Primers**

**Table 2: Comparison of AUC Between groups**

| Dependent Variable: AUC | | | | | | |
| --- | --- | --- | --- | --- | --- | --- |
| Post Hoc: Tamhane | | | | | | |
| (I) group | (J) group | Mean Difference (I-J) | Std. Error | Sig. | 95% Confidence Interval | |
|  |  |  |  |  | Lower Bound | Upper Bound |
| EAE+DMSo5% &Saline | EAE+ Melatonin 0.1mg/Kg | 15.92308^*^ | 1.34808 | .0000000263 | 11.5529 | 20.2932 |
|  | EAE+ Melatonin 1mg/Kg | 13.34615^*^ | 1.24223 | .0000000619 | 9.3483 | 17.3440 |
|  | EAE+ Melatonin 5mg/Kg | 14.26923^*^ | 1.81919 | .0004286085 | 7.3940 | 21.1445 |
|  | EAE+ Melatonin 10 mg/Kg | 14.61538^*^ | 1.93980 | .0000297468 | 8.1704 | 21.0604 |
| EAE+ Melatonin 0.1mg/Kg | EAE+DMSo5% &Saline | -15.92308^*^ | 1.34808 | .0000000263 | -20.2932 | -11.5529 |
|  | EAE+ Melatonin 1mg/Kg | -2.57692 | 1.68252 | .776 | -7.7655 | 2.6116 |
|  | EAE+ Melatonin 5mg/Kg | -1.65385 | 2.14410 | .998 | -8.7729 | 5.4652 |
|  | EAE+ Melatonin 10 mg/Kg | -1.30769 | 2.24734 | 1.000 | -8.3351 | 5.7197 |
| EAE+ Melatonin 1mg/Kg | EAE+DMSo5% &Saline | -13.34615^*^ | 1.24223 | .0000000619 | -17.3440 | -9.3483 |
|  | EAE+ Melatonin 0.1mg/Kg | 2.57692 | 1.68252 | .776 | -2.6116 | 7.7655 |
|  | EAE+ Melatonin 5mg/Kg | .92308 | 2.07918 | 1.000 | -6.0858 | 7.9320 |
|  | EAE+ Melatonin 10 mg/Kg | 1.26923 | 2.18549 | 1.000 | -5.6115 | 8.1500 |
| EAE+ Melatonin 5mg/Kg | EAE+DMSo5% &Saline | -14.26923^*^ | 1.81919 | .0004286085 | -21.1445 | -7.3940 |
|  | EAE+ Melatonin 0.1mg/Kg | 1.65385 | 2.14410 | .998 | -5.4652 | 8.7729 |
|  | EAE+ Melatonin 1mg/Kg | -.92308 | 2.07918 | 1.000 | -7.9320 | 6.0858 |
|  | EAE+ Melatonin 10 mg/Kg | .34615 | 2.55786 | 1.000 | -7.7876 | 8.4799 |
| EAE+ Melatonin 10 mg/Kg | EAE+DMSo5% &Saline | -14.61538^*^ | 1.93980 | .0000297468 | -21.0604 | -8.1704 |
|  | EAE+ Melatonin 0.1mg/Kg | 1.30769 | 2.24734 | 1.000 | -5.7197 | 8.3351 |
|  | EAE+ Melatonin 1mg/Kg | -1.26923 | 2.18549 | 1.000 | -8.1500 | 5.6115 |
|  | EAE+ Melatonin 5mg/Kg | -.34615 | 2.55786 | 1.000 | -8.4799 | 7.7876 |
| *. The mean difference is significant at the 0.05 level. | | | | | | |

**Table 3: Comparison of Cumulative Score Between groups**

| Dependent Variable: Cumulative Score | | | | | | |
| --- | --- | --- | --- | --- | --- | --- |
| Post Hoc Test: Tamhane | | | | | | |
| (I) group | (J) group | Mean Difference (I-J) | Std. Error | Sig. | 95% Confidence Interval | |
|  |  |  |  |  | Lower Bound | Upper Bound |
| EAE+DMSo5% &Saline | EAE+ Melatonin 0.1mg/Kg | 17.84615^*^ | 1.48862 | .00000003 | 13.0022 | 22.6901 |
|  | EAE+ Melatonin 1mg/Kg | 14.76923^*^ | 1.39207 | .00000011 | 10.2649 | 19.2736 |
|  | EAE+ Melatonin 5mg/Kg | 15.75000^*^ | 2.00006 | .00044755 | 8.1584 | 23.3416 |
|  | EAE+ Melatonin 10 mg/Kg | 16.30769^*^ | 2.14949 | .00002980 | 9.1489 | 23.4665 |
| EAE+ Melatonin 0.1mg/Kg | EAE+DMSo5% &Saline | -17.84615^*^ | 1.48862 | .00000003 | -22.6901 | -13.0022 |
|  | EAE+ Melatonin 1mg/Kg | -3.07692 | 1.88788 | .709 | -8.8969 | 2.7430 |
|  | EAE+ Melatonin 5mg/Kg | -2.09615 | 2.37202 | .993 | -9.9622 | 5.7699 |
|  | EAE+ Melatonin 10 mg/Kg | -1.53846 | 2.49931 | 1.000 | -9.3536 | 6.2767 |
| EAE+ Melatonin 1mg/Kg | EAE+DMSo5% &Saline | -14.76923^*^ | 1.39207 | .00000011 | -19.2736 | -10.2649 |
|  | EAE+ Melatonin 0.1mg/Kg | 3.07692 | 1.88788 | .709 | -2.7430 | 8.8969 |
|  | EAE+ Melatonin 5mg/Kg | .98077 | 2.31265 | 1.000 | -6.7813 | 8.7428 |
|  | EAE+ Melatonin 10 mg/Kg | 1.53846 | 2.44304 | 1.000 | -6.1423 | 9.2192 |
| EAE+ Melatonin 5mg/Kg | EAE+DMSo5% &Saline | -15.75000^*^ | 2.00006 | .00044755 | -23.3416 | -8.1584 |
|  | EAE+ Melatonin 0.1mg/Kg | 2.09615 | 2.37202 | .993 | -5.7699 | 9.9622 |
|  | EAE+ Melatonin 1mg/Kg | -.98077 | 2.31265 | 1.000 | -8.7428 | 6.7813 |
|  | EAE+ Melatonin 10 mg/Kg | .55769 | 2.83387 | 1.000 | -8.4500 | 9.5654 |
| EAE+ Melatonin 10 mg/Kg | EAE+DMSo5% &Saline | -16.30769^*^ | 2.14949 | .00002980 | -23.4665 | -9.1489 |
|  | EAE+ Melatonin 0.1mg/Kg | 1.53846 | 2.49931 | 1.000 | -6.2767 | 9.3536 |
|  | EAE+ Melatonin 1mg/Kg | -1.53846 | 2.44304 | 1.000 | -9.2192 | 6.1423 |
|  | EAE+ Melatonin 5mg/Kg | -.55769 | 2.83387 | 1.000 | -9.5654 | 8.4500 |
| *. The mean difference is significant at the 0.05 level. | | | | | | |

**Figure 1: Calculating AUC with R**


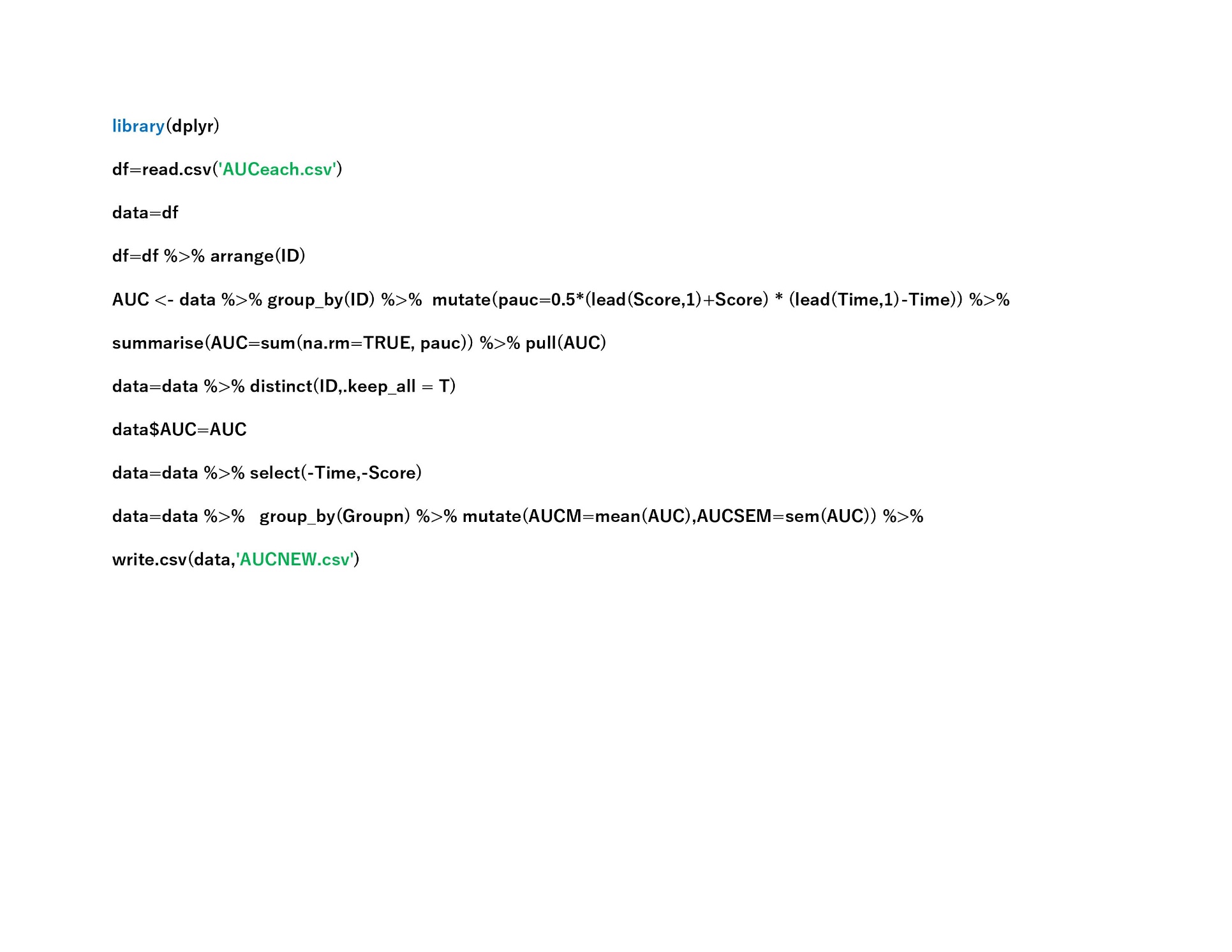

Supplement: Supplementary file 1 — Supplementary Information. [file 41598_2022_20164_MOESM1_ESM.docx]
